# PRESIDENTIAL SYMPOSIUM: NUTRITION, METABOLISM, AND AGING

**DOI:** 10.1093/geroni/igac059.360

**Published:** 2022-12-20

**Authors:** Rozalyn Anderson

## Abstract

Aging is associated with increased risk for a host of non-communicable diseases and disorders including diabetes, cardiovascular disease, cancer, and neurodegeneration. Although the underlying basis for this shared risk as a function of aging is not known, numerous diseases of aging have an established metabolic component. Data from preclinical and basic research indicate that the pace of aging is malleable, and studies in short-lived species have shown the ability of genetic and nutritional interventions to not only positively impact longevity but also to prolong health into older age. This symposium, “Nutrition, Metabolism, & Aging”, features internationally renowned aging research scientists whose work focuses on how nutrition and metabolism intersect with aging biology. We will hear from John Speakman from the University of Aberdeen who will present his research on “Metabolism and Extended Longevity”; Leanne Redman from the Pennington Biomedical Research Center whose work in humans will be described in her talk on “Dietary Interventions for Healthy Aging”; Holly Brown-Borg from University of North Dakota will discuss endocrine modulation of aging in her talk “Somatotropic Signaling in Health and Longevity”; and Valter Longo from the University of Southern California who will discuss the latest findings from his research in mice and humans in his talk “Periodic Fasting in Aging and Disease". Attendees will learn about nutrition and metabolism and how these cues are interwoven to regulate aging, impact health, and enhance longevity.

